# Thyrotoxicosis and Choledocholithiasis Masquerading as Thyroid Storm

**DOI:** 10.1155/2017/9454698

**Published:** 2017-08-23

**Authors:** Christian L. Horn, Patricia A. Short

**Affiliations:** Madigan Army Medical Center, Tacoma, WA, USA

## Abstract

A 26-year-old female, thirteen months postpartum, presented to the emergency department for four weeks of epigastric abdominal pain, pruritus, new onset jaundice, and 11.3 kgs (25 lbs) unintentional weight loss. On examination, she was afebrile, tachycardic, alert, and oriented and had jaundice with scleral icterus. Labs were significant for undetectable TSH, FT4 that was too high to measure, and elevated total bilirubin, direct bilirubin, alkaline phosphatase, and transaminases. Abdominal ultrasound revealed cholelithiasis without biliary ductal dilation. Treatment for presumed thyroid storm was initiated. Further work-up with magnetic resonance cholangiopancreatography (MRCP) revealed an obstructing cholelith within the distal common bile duct. With the presence of choledocholithiasis explaining the jaundice and abdominal pain, plus the absence of CNS alterations, the diagnosis of thyroid storm was revised to thyrotoxicosis complicated by choledocholithiasis. Endoscopic retrograde cholangiopancreatogram (ERCP) with sphincterotomy was performed to alleviate the biliary obstruction, with prompt symptomatic improvement. Thyroid storm is a rare manifestation of hyperthyroidism with a high rate of morbidity and mortality. The diagnosis of thyroid storm is based on clinical examination, and abnormal thyroid function tests do not correlate with disease severity. Knowledge of the many manifestations of thyroid storm will facilitate a quick and accurate diagnosis and treatment.

## 1. Introduction

Thyroid storm is a rare, life-threatening condition marked by multisystem dysfunction in the setting of thyrotoxicosis. Hallmark manifestations of thyroid storm include hyperthermia, central nervous system (CNS) dysfunction, cardiovascular aberrations, and gastrointestinal abnormalities. While laboratory evidence of thyrotoxicosis is helpful in the diagnosis of thyroid storm, the importance of clinical examination and judgment is crucial in distinguishing thyrotoxicosis from thyroid storm. Several diagnostic criteria have been established to aid in differentiating thyrotoxicosis from thyroid storm. It is important to understand that the incidence of thyroid storm in the hospitalized population is noticeably low; however the mortality rate associated with this disease process is alarmingly high. Early identification of this challenging diagnosis will decrease the time to appropriate treatment and help to prevent disease morbidity and mortality. We present a case that highlights the challenges a general internist may face in making the appropriate diagnosis of thyroid storm in a hospitalized thyrotoxic patient.

## 2. Case Presentation

A 26-year-old female presented to the emergency department due to a four-week history of intermittent sharp epigastric abdominal pain. The abdominal pain had been occurring intermittently over the course of the previous four months, typically occurring in the evenings. The pain started very suddenly, reaching peak intensity about one hour after it started. She denied association of the pain with eating meals. Review of symptoms revealed that the week prior to admission, she developed diffuse pruritus associated with a yellowish discoloration of her skin. She also admitted to an unintentional 11.3-kilogram (25-pound) weight loss over the previous three months despite polyphagia over the same time period. Notably, she denied tremors, excessive sweating, heat or cold intolerance, diarrhea, or constipation. She had no previous medical or surgical history and was not taking any medications; however she had given birth to her first child thirteen months prior to presentation.

While in the emergency department, she demonstrated persistent tachycardia, with a heart rate reaching a peak of 134 beats per minute (bpm), but had stable blood pressure at 131/81 mmHg. She was afebrile and without respiratory distress. On physical examination, she was alert and oriented to person, place, and time, and answering questions appropriately. She had scleral icterus, mild lid lag, and sublingual jaundice. Her heart was tachycardic but had a regular rhythm without murmur, rubs, or gallops, with clear lung fields bilaterally. Abdominal exam failed to elicit tenderness, rebound pain, involuntary guarding, or Murphy's sign.

Laboratory investigation revealed a normal complete blood count and basic metabolic panel. Hepatic function panel was notable for a total bilirubin of 4.2 mg/dL (reference range: 0-1 mg/dL), direct bilirubin of 3.8 mg/dL (reference range: 0–0.3 mg/dL), indirect bilirubin of 0.4 mg/dL (reference range: 0-1.0 mg/dL), alkaline phosphatase of 127 U/L (reference range: 35–104 U/L), alanine aminotransferase of 106 U/L (reference range: 0–33 U/L), and aspartate aminotransferase of 79 U/L (reference range: 0–32 U/L). Thyroid stimulating hormone (TSH) level was less than 0.01 mcIU/mL (reference range: 0.27–4.32 mcIU/mL) and her free T4 (FT4) was greater than 7.77 ng/dL (reference range: 0.58–1.64 ng/dL), higher than could be measured. She underwent a right-upper-quadrant ultrasound which showed cholelithiasis with a gallbladder wall thickness of 3 mm and a common bile duct diameter of 5 mm, but no pericholecystic fluid. She was given IV Propranolol to help control her heart rate and was admitted to the hospital for further management.

**Figure 1:**
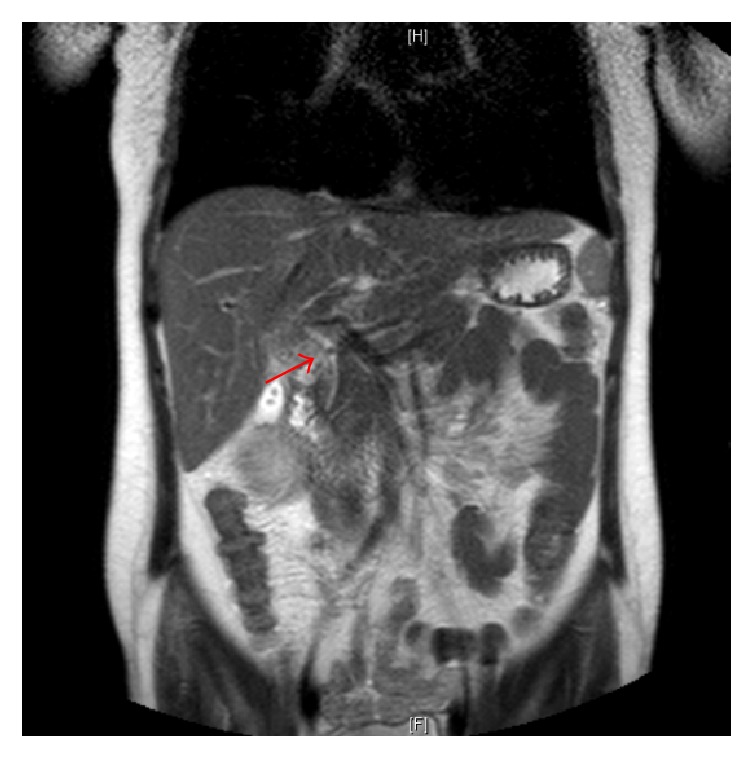
MRCP showing a 4 mm (arrow) obstructing gallstone in the distal common bile duct.

**Figure 2:**
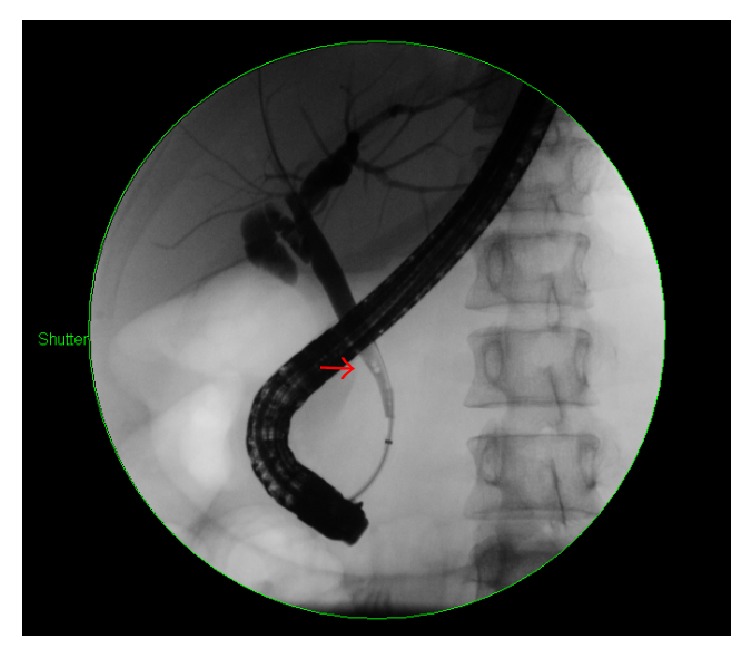
ERCP image of the gallstone (arrow) prior to removal.

Given her severe tachycardia, unexplained jaundice, nausea, and vomiting, there was a high suspicion for thyroid storm. She was initially started on Propranolol 20 mg four times daily, Methimazole 20 mg three times daily, and Prednisone 40 mg daily due to concern for thyroid storm. Further imaging with magnetic resonance cholangiopancreatography (MRCP) showed the presence of a 4-millimeter obstructing gallstone within the distal common bile duct, without biliary duct dilation (Figure 1). She underwent endoscopic retrograde cholangiopancreatography (ERCP) with sphincterotomy and removal of her gallstone, with subsequent improvement in her liver enzyme abnormalities (Figure 2). The Prednisone was stopped after an explanation for her jaundice and gastrointestinal symptoms was established, and it was determined that she was not in thyroid storm.

Her heart rate was difficult to control during the beginning of her hospital stay and required dose escalation of Propranolol, ultimately to 80 mg four times daily. Transthoracic echocardiogram with Doppler and color flow Doppler demonstrated normal left ventricular size and function with an ejection fraction of 55%. Ultrasound of her thyroid gland revealed a heterogenous stroma consistent with Grave's disease. The diagnosis of Grave's disease was confirmed by an elevated activity of thyroid stimulating hormone receptor antibody, up to 639% of basal activity (reference range <140% basal activity). Cholecystectomy was deferred until her Grave's disease was better controlled.

## 3. Discussion

This case demonstrates the challenge of appropriately diagnosing thyroid storm in a hospitalized thyrotoxic patient. Thyroid storm represents the most severe manifestations of thyrotoxicosis, marked by multisystem dysfunction. While the disease itself is uncommon, the morbidity and mortality associated with it were severe enough that prompt recognition is necessary.

The importance of distinguishing between simple thyrotoxicosis and thyroid storm is emphasized by the high mortality rate associated with thyroid storm. Burch and Wartofsky originally estimated the mortality rate to be between 20 and 50% when they published their diagnostic criteria in 1993; however more recent estimates place the rate at 10–30% [1, 3]. In 2012, the Japan Thyroid Association performed a nationwide survey of thyroid storm in Japan, which showed the incidence of disease to be only 1-2% of hospitalized thyrotoxic patients, but the mortality rate was 11% based on their diagnostic criteria for definite thyroid storm [2, 3].

The delineation of thyrotoxicosis and thyroid storm is controversial and difficult to establish but is essential in order to initiate appropriate treatment. Often the level of measured thyroid hormone can be identical in cases of both simple thyrotoxicosis and thyroid storm, making it necessary to follow established criteria for differentiating between the two disease processes [4–6]. Burch and Wartofsky created the first widely accepted, and still widely utilized, clinical scoring system for establishing the diagnosis of thyroid storm (Table 1). They assigned a point value to clinical manifestations of the disease, including neurologic manifestations, temperature elevations, tachycardia, atrial fibrillation, gastrointestinal/hepatic symptoms, and precipitant history. Using these clinical manifestations, they created cut-offs for minimal point score for thyroid storm [1]. For patients that meet the minimal cut-off, the provider can make the diagnosis of thyroid storm with relative confidence.

While Burch and Wartofsky's scoring system is still widely utilized, it is not the only available diagnostic criteria for thyroid storm. The diagnostic criteria established by the Japan Thyroid Association are a clinical tool that does not rely on assigning point values to manifestations of the disease (Table 2). Instead, the Japan Thyroid Association created two different clinical entities encompassing thyroid storm, TS1 represents definite thyroid storm, and TS2 defines suspected thyroid storm. In order to qualify for TS1, patients need to have laboratory evidence of thyrotoxicosis, in addition to certain combinations of clinical manifestations. TS2 diagnostic criteria use the same clinical manifestations; however the requirements are less stringent than TS1 and also do not necessarily require laboratory evidence of thyrotoxicosis [2].

Our patient provides an excellent example in the difficulties of navigating the diagnosis of thyroid storm. When she presented to the emergency department, she had multiple signs and symptoms consistent with a diagnosis of thyroid storm and a recent precipitant history of parturition. Her symptoms included tachycardia up to 134 bmp, nausea, vomiting, abdominal pain, and unexplained jaundice. Her abdominal pain was not classic for biliary obstruction, because her pain was not associated with oral intake, and while elevations of direct bilirubin is consistent with a cholestatic liver injury and potential biliary obstruction, a cholestatic liver enzyme pattern is also commonly observed in thyroid storm [7]. Additionally, ultrasonographic imaging in the emergency department failed to show the presence of ductal dilation or obstructing cholelith, so at that time her symptoms were not thought to be caused by biliary obstruction.

Based on Burch and Wartofsky's criteria for thyroid storm, we calculated an initial score for our patient of 60 from her tachycardia (20 points), nausea, vomiting, and abdominal pain (10 points), unexplained jaundice (20 points), and precipitant history of giving birth (10 points). A score of 60 on the Burch and Wartofsky scale is highly suggestive of thyroid storm. She did not receive points for thermoregulatory dysfunction, atrial fibrillation, heart failure, or central nervous system (CNS) effects. Once the diagnosis of choledocholithiasis was established via MRCP, we were adequately able to explain the jaundice and gastrointestinal symptoms and revised the Burch and Wartofsky score to 35, suggestive of impending thyroid storm.

Evaluation of our patient using the criteria recently established by the Japan Thyroid Association might have changed initial management in our patient. Given the combination of signs and symptoms upon initial presentation, specifically laboratory evidence of hyperthyroidism, tachycardia greater than 130 bpm, and GI/hepatic manifestations, our patient would only have only fulfilled criteria for the first combination of TS2, indicating a diagnosis of suspected thyroid storm.

One major factor that was absent in our patient which strongly argues against a diagnosis of thyroid storm is neurologic alterations. Neurologic findings in thyroid storm are nearly universal, with the most prominent features being restlessness, somnolence/lethargy, and delirium. The estimated prevalence of neurological involvement in thyroid storm from the literature is 64.2%; however, among patients diagnosed with TS1 by the Japanese Thyroid Association, the prevalence is 84.4% (2.7% among patients with TS2) [2]. The absence of CNS alterations is more consistent with a picture of thyrotoxicosis.

Management of thyroid storm and uncontrolled hyperthyroidism overall incorporates a similar approach, including treatment with beta-adrenergic blockers and antithyroid hormones; however treatment of thyroid storm also includes adding inorganic iodine or corticosteroids and aggressively treating manifestations of the disease. According to American Thyroid Association (ATA) and American Association of Clinical Endocrinologists (AACE) 2011 guidelines, overt hyperthyroidism can be treated with either I^131^ therapy, antithyroid medications, or thyroidectomy. Inorganic iodine works by blocking new thyroid hormone synthesis and release from the thyroid gland and can rapidly lower circulating thyroid hormone levels. Despite the utility of inorganic iodine, it must be given an hour after starting antithyroid hormone therapy due to the risk of exacerbating the thyrotoxic state [1, 8]. The benefit of glucocorticoids in thyroid storm includes blocking peripheral conversion of T4 to T3 and also is used as prophylaxis in the event of concomitant adrenal insufficiency [8]. In our patient's case, discovery of her choledocholithiasis occurred before inorganic iodine could be initiated for thyroid storm and is not indicated in thyrotoxicosis; therefore this treatment modality was not used.

## 4. Conclusion

In conclusion, thyroid storm has a low incidence; however, it is associated with significant morbidity and mortality if left untreated. Thyroid storm cannot be differentiated from thyrotoxicosis based on lab abnormalities alone, and clinical manifestations must be considered. In order to make the appropriate diagnosis, a clinician needs to have a high index of suspicion, making the diagnosis based on clinical judgment. Different diagnostic tools have been established to aid in the process of diagnosis; however, they should not be used as a substitute for sound clinical reasoning. By rapidly establishing the diagnosis of thyroid storm, physicians will decrease the time of treatment and reduce the morbidity and mortality associated with this disease.

## Figures and Tables

**Table 1:** Diagnostic criteria for thyroid storm established by Burch and Wartofsky. A score below 25 is unlikely to represent thyroid storm, a score between 25 and 44 is suggestive of impending thyroid storm, and a score greater than or equal to 45 is highly suggestive of thyroid storm.

Diagnostic criteria for thyroid storm adapted from Burch and Wartofsky [1]			
Thermoregulatory dysfunction		Cardiovascular dysfunction	
Temperature (°F)		Tachycardia (heart rate)	
99–99.9	5	90–109	5
100–100.9	10	110–119	10
101–101.9	15	120–129	15
102–102.9	20	130–139	20
103–103.9	25	≥140	25
≥104.0	30	Congestive heart failure	
*Central nervous system effects*		Absent	0
Absent	0	Mild	5
Mild	10	Pedal edema	
Agitation		Moderate	10
Moderate	20	Bibasilar rales	
Delirium, psychosis, extreme lethargy		Severe	15
Severe	30	Pulmonary edema	
Seizure, coma		Atrial fibrillation	
*Gastrointestinal-hepatic dysfunction*		Absent	0
Absent	0	Present	10
Moderate	10	*Precipitant history* ^*∗*^	
Diarrhea, nausea/vomiting, abdominal pain		Negative	0
Severe	20	Positive	10
Unexplained jaundice			

^*∗*^Precipitant history includes thyroid surgery, withdrawal of antithyroid drug therapy, radioiodine therapy, vigorous thyroid palpation, iodinated contrast dyes, nonthyroidal surgery, infection, cerebrovascular accident, pulmonary thromboembolism, parturition, diabetic ketoacidosis, emotional stress, and trauma.

**Table 2:** Diagnostic criteria of thyroid storm established by the Japan Thyroid Association.

Final criteria for the diagnosis of thyroid storm adapted from Akamizu et al. [2]		
Grade of TS	Combinations of features	Requirements for diagnosis
TS1	First combination	Thyrotoxicosis and at least one CNS manifestation, and one of the following: fever, tachycardia, CHF, or GI/hepatic manifestations

TS1	Alternate combination	Thyrotoxicosis and at least three combinations of fever, or tachycardia, or CHF< or GI/hepatic manifestations

TS2	First combination	Thyrotoxicosis and a combination of two of the following: fever or tachycardia or CHF or GI/hepatic manifestations

TS2	Alternate combination	Patients who meet the diagnostic criteria for TS1 except that serum FT3 or FT4 values are not available but whose data before or after the episode suggest that they are thyrotoxic at the time of TS

*Definitions.* (i) TS1: definite thyroid storm; TS2: suspected thyroid storm; (ii) thyrotoxicosis: elevated FT3 or FT4; (iii) CNS manifestations: restlessness, delirium, mental aberration/psychosis, somnolence/lethargy, convulsion, and coma including a score of 1 or higher on the Japan Coma Scale (JCS) or 14 or lower on the Glasgow Coma Scale (GCS); (iv) fever: 38°C or higher; (v) tachycardia: ≥130 beats/min (arrhythmias such as atrial fibrillation are evaluated by measuring the heart rate); (vi) CHF: the patient presenting with severe symptoms such as pulmonary edema, moist rales for more than half the lung field, or cardiogenic shock. The patient's CHF is categorized as Class IV by the NYHA classification or Class III or higher by the Killip classification; (vii) GI/hepatic manifestations: the patient presenting with nausea, vomiting, diarrhea, or a bilirubin of >3 mg/dL.
